# Development and validation of a low-cost, wearable near-infrared spectroscopy system for quantifying tissue water fraction in the human brain

**DOI:** 10.1117/1.JBO.31.10.106006

**Published:** 2026-10-28

**Authors:** Kuan Cheng Wu, Marco Renna, Nikola Otic, Zachary Starkweather, Olivia S. Kierul, Christopher Smith, Maria Angela Franceschini, Mitchell B. Robinson

**Affiliations:** Athinoula A. Martinos Center for Biomedical Imaging, Massachusetts General Hospital, Harvard Medical School, Charlestown, Massachusetts, United States

**Keywords:** near-infrared spectroscopy, cerebral water fraction, wearable device, cerebral hemodynamics, glymphatic system, light-emitting diode

## Abstract

**Significance:**

Cerebral water fraction is physiologically relevant to brain fluid dynamics, glymphatic function, cerebral edema, and dehydration, yet it is not routinely measured in wearable neuroimaging.

**Aim:**

We aimed to develop and validate a low-cost, wearable, light-emitting diode (LED)-based continuous-wave near-infrared spectroscopy system capable of concurrently monitoring changes in cerebral hemoglobin concentration and water fraction from the forehead.

**Approach:**

Our device, called the GS-FlexNIRS, contains nine LEDs spanning 740 to 1050 nm mounted on a flexible printed circuit board. Three of these wavelengths (i.e., 910, 940, and 1050 nm) fall within the water absorption window. A wavelength-weighted fitting algorithm incorporates measured LED emission spectra and photodiode responsivity to enable accurate chromophore recovery despite spectrally broad LED illumination. Performance was characterized in liquid phantoms and in five healthy participants during respiratory maneuvers.

**Results:**

The system achieved a dynamic range of ∼100  dB, gain linearity ($R^{2}>0.99997$), and signal-to-noise ratio of 67 to 90 dB. Phantom experiments confirmed independent tracking of hemoglobin deoxygenation and water fraction changes. Hemoglobin responses in human measurements were consistent with prior work.

**Conclusions:**

The GS-FlexNIRS (∼$350/unit) and its wearable design may support future longitudinal and naturalistic monitoring of cerebral hemodynamics and brain fluid dynamics, enabling investigation in sleep, glymphatic research, and cerebral edema monitoring.

## 1 Introduction

Water is the dominant component of biological tissue and comprises ∼60% of the human body.^1^ The dynamics of water transport are directly involved in key physiological processes, including blood volume regulation, interstitial fluid transport, and cerebrospinal fluid (CSF) flow.^2^^,^^3^ Alterations in tissue water content are associated with conditions such as edema, dehydration, and impaired fluid clearance.^3^^,^^4^ In the brain, water dynamics are closely linked to CSF flow and CSF-interstitial fluid exchange, and it is theorized that these CSF dynamics aid in cerebral metabolic waste clearance^4^^–^^6^ through what has been termed the glymphatic system (“glial lymphatic” system). Increases in CSF flux and large CSF oscillations have been observed during non-rapid eye movement sleep in association with slow electrophysiological oscillations, vascular fluctuations, and autonomic activity thought to drive increased clearance during sleep.^7^^,^^8^ Impaired clearance function has been implicated in aging and neurodegenerative diseases through the accumulation of metabolites such as amyloid-β and tau.^9^^,^^10^

Despite its physiological relevance, assessment of CSF-related fluid dynamics in humans remains challenging and is therefore not routinely measured, particularly in longitudinal settings.^11^ Current investigations of brain fluid transport and glymphatic function in humans rely primarily on magnetic resonance imaging (MRI), using either exogenous tracers^12^ or endogenous contrasts.^7^^,^^13^^,^^14^ Although MRI has provided critical insights, its cost, technical complexity, and lack of portability limit its use for continuous, long-duration overnight measurements and preclude monitoring in naturalistic environments such as sleep at home.^15^

Alternatively, bioimpedance spectroscopy has shown potential for noninvasive estimation of total body water content and its intracellular and extracellular fluid compartments.^16^ Brain parenchymal impedance measurements obtained during sleep using implanted intracranial leads^17^ and noninvasive earpieces^18^ have shown sleep-associated impedance reductions. Although these reductions in tissue impedance in humans are consistent with an increase in extracellular space volume observed in animal sleep models as well as in imaging of human brain changes in contrast-enhanced MRI-determined clearance,^6^ the currently demonstrated temporal resolution of these impedance approaches^17^^,^^18^ was insufficient to resolve the ∼20-s CSF oscillations observed during sleep.^7^ To capture these coupled dynamics, a noninvasive, wearable method with direct sensitivity and ability to continuously and quickly sample tissue water content is needed.

Over the past four decades, near-infrared spectroscopy (NIRS) has been widely used for noninvasive monitoring of cerebral physiology.^19^^,^^20^ Traditionally, continuous-wave NIRS (CW-NIRS) has been used to quantify changes in oxyhemoglobin (HbO) and deoxyhemoglobin (HbR) by measuring wavelength-dependent light attenuation at two or more wavelengths within the 650 to 950 nm range^21^ and is well suited for wearable applications.^22^ In practice, however, CW-NIRS is typically configured with wavelengths≤850  nm, where water absorption is comparatively weak relative to the contributions of HbO and HbR,^23^ limiting its applicability for studying fluid dynamic processes such as those driving CSF-mediated cerebral waste clearance. To capture these dynamics, water-sensitive NIRS systems based on silicon detectors employ wavelengths in the spectral window between ∼900 and 1100 nm.^24^ Previous studies have demonstrated water measurements using broadband NIRS^25^ and laser-based CW-NIRS,^26^^,^^27^ but these systems are often bulky, expensive, or not optimized for wearable applications. Although water absorption increases further beyond the 1100 nm range,^28^^,^^29^ in principle providing increased water sensitivity, the resulting increased effective attenuation coefficient significantly reduces photon penetration depth in the diffuse optical regime, limiting sensitivity to cortical tissue.^30^ This contrasts with multiphoton microscopy^31^ and optical coherence tomography (OCT),^32^ where decreased scattering at longer wavelengths instead enhances penetration depth.^24^

Here, we describe a low-cost, light-emitting diode (LED)-based, wearable, wireless CW-NIRS device, the Glymphatic System FlexNIRS (GS-FlexNIRS),^33^ with wavelengths in the 900 to 1100 nm water absorption window, enabling simultaneous measurement of hemoglobin dynamics and tissue water fraction. LEDs are well suited for wearable NIRS due to their low cost, noncoherent emission, improved safety, and reduced sensitivity to speckle noise.^34^ Although largely beneficial, a key limitation of LEDs is their broad emission bandwidth, which reduces spectral specificity for chromophores with sharp absorption features, including water near 980 nm. To address this limitation, we utilize a spectrum-conscious framework, developed previously,^35^ to enable accurate recovery of water fraction despite the spectrally broad LED emission.

We characterized the device and validated its performance using two complementary liquid phantom experiments, confirming accurate tracking of hemoglobin deoxygenation and sensitivity to water fraction changes independently of hemoglobin. Feasibility was also assessed in a pilot study of healthy participants during controlled respiratory maneuvers. Taken together, these results support the GS-FlexNIRS as a low-cost platform for future longitudinal monitoring of cerebral hemodynamics and brain fluid dynamics in naturalistic settings.

## 2 Materials and Methods

### 2.1 GS-FlexNIRS System Overview

The GS-FlexNIRS system consists of an optical sensor and a compact control module integrated on a single flexible printed circuit board (PCB) [Fig. 1(a)]. This design allows the device to conform to the curvature of the forehead, improving skin contact and promoting stable optical coupling. A photograph of the populated flexible PCB prior to encapsulation is shown in Fig. 1(b), and the encapsulated device is shown in Fig. 1(c). The system is powered by a medical-grade rechargeable 600 mAh lithium-polymer (Li-Po) battery (LP503040JH, Jauch Quartz GmbH, Germany) connected via a magnetic pogo-pin connector, enabling easy battery replacement. The electronics and flexible PCB were coated with a waterproofing layer (Plasti Dip, Plasti Dip International, Blaine, Minnesota, United States) and subsequently encapsulated in a low-profile silicone elastomer using a 3D-printed mold. The device is secured to the forehead with an elastic headband, which also holds the battery; placement and attachment are described in Sec. S1 of the Supplementary Material.

**Fig. 1:**
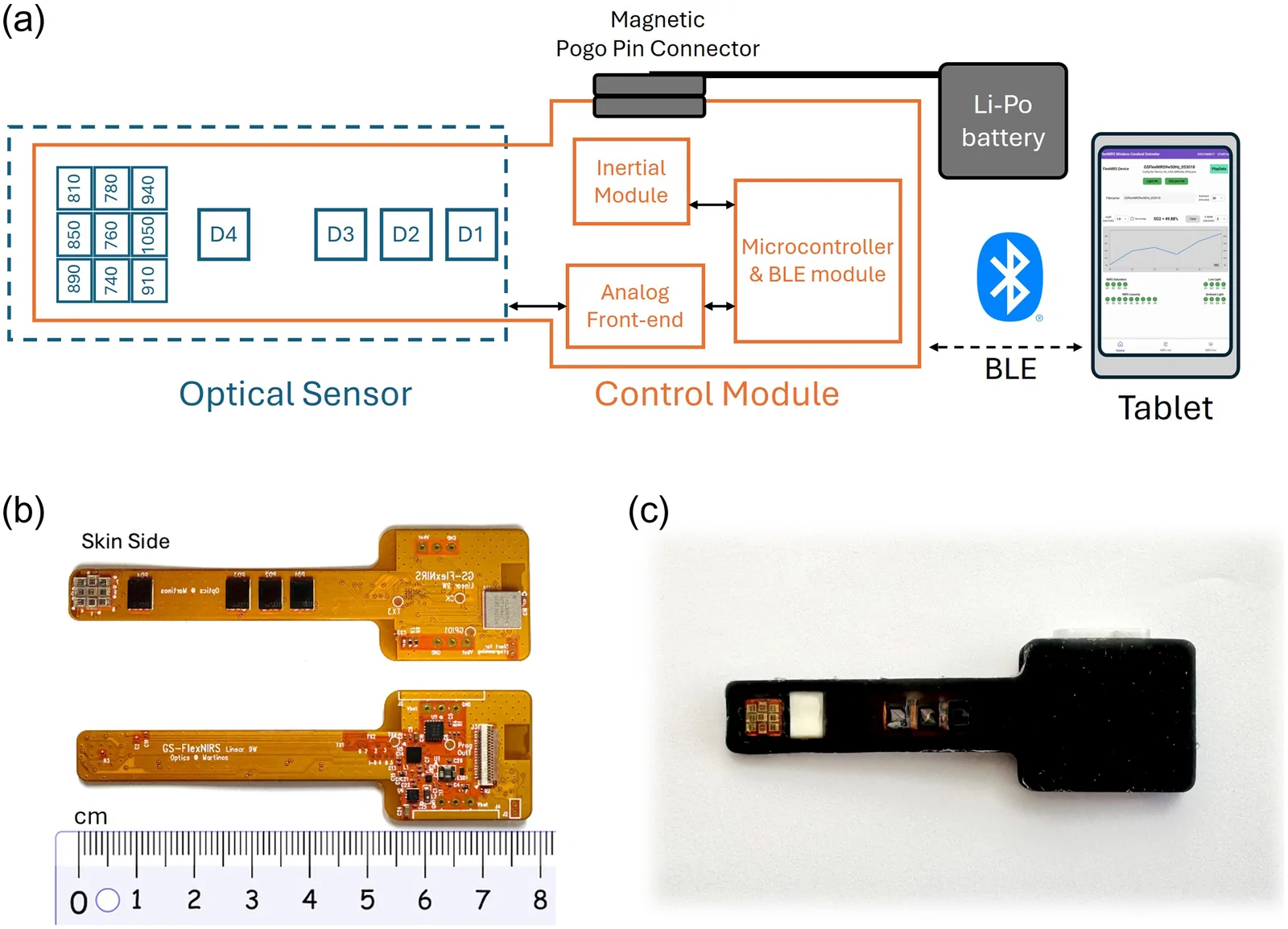
(a) Simplified block diagram of the GS-FlexNIRS system. The optical sensor comprises a 3×3 array of nine LEDs (nominal wavelengths indicated) and four photodiode detectors (D1 to D4). The control module integrates an analog front-end, inertial module, and microcontroller with Bluetooth® Low Energy (BLE) for wireless data transmission. (b) Photograph of the flexible PCB with components populated, prior to encapsulation. The skin-contacting side is labeled. A ruler is shown for scale (unit: centimeters). (c) Photograph of the encapsulated GS-FlexNIRS device. The white region is a thin scattering film placed over the short-separation photodiode to attenuate the incident light and prevent detector saturation.

The optical sensor consists of nine high-power LED sources (EDCC series, Marubeni, Tokyo, Japan) arranged in a 3×3 array and four photodiode (PD) detectors (VEMD5080X01, Vishay Intertechnology, Inc., Malvern, Pennsylvania, United States). The wavelengths (740, 760, 780, 810, 850, 890, 910, 940, and 1050 nm) were selected to balance commercial availability with broad spectral coverage across the near-infrared window, providing sensitivity to the main tissue chromophores: oxyhemoglobin, deoxyhemoglobin, and water. LED positions within the 3×3 array were selected to balance tissue attenuation and source–detector distance. The small differences in LED position were accounted for in post-processing (Sec. 2.4). To support the higher forward voltage of some of the LED modules (dual-junction LEDs within the EDCC series), similar to the design presented previously,^35^ a DC/DC converter was used to translate the battery voltage to the required 5.25 V driver voltage. Three PDs are positioned at long source–detector separations (SDS) of ∼2.5, 3.0, and 3.5 cm from the center of the LED array. A fourth PD is placed ∼0.8  cm as the short-separation channel. A thin scattering film was placed over the short-separation PD to prevent light saturation during measurements [Fig. 1(c)]. Optical signal acquisition and LED driving are handled by a dedicated analog front-end (AFE) integrated circuit (AFE4460, Texas Instruments, Dallas, Texas, United States), which supports up to 32 independently driven LEDs and parallel sampling of up to four PDs across 16 programmable phases. To improve the signal-to-noise ratio, the two current drivers of the AFE were combined to drive each LED, achieving a maximum current of 334 mA. This configuration supports the nine-LED, four-detector GS-FlexNIRS architecture.

A three-axis accelerometer (ADXL344, Analog Devices, Wilmington, Massachusetts, United States) is mounted on the flexible PCB, enabling detection of participant movement for motion artifact identification and rejection during data processing. A thermistor (NTCG063JF103FTDS, TDK Corporation, Tokyo, Japan) mounted on the opposite side of the flexible PCB beneath the LED array monitors LED temperature during operation.

System operation is coordinated by a Bluetooth® Low Energy (BLE) microcontroller (BGM220S, Silicon Labs, Austin, Texas, United States), which programs the AFE, acquires optical, temperature and inertial data via SPI (AFE) and $I^{2}C$ (accelerometer), and transmits the data wirelessly using BLE version 5.4. Data transmitted via BLE are received and displayed in real time using a custom cross-platform application (Flutter, Google, Mountain View, California, United States), which performs device configuration, live signal monitoring, and data logging on both Android and iOS (Sec. S4 of the Supplementary Material). Collected data were post-processed using custom scripts developed in MATLAB (MathWorks, Natick, Massachusetts, United States).

### 2.2 Acquisition Sequence

Data acquisition is organized into repeating cycles of 16 phases, controlled by the AFE, with an additional ambient acquisition. Each phase lasts 117  μs, during which a single LED is activated while signals from all four PDs are recorded simultaneously. Including analog-to-digital conversion and ambient light sampling, the full acquisition cycle runs at 100 Hz. The LED phase sequence is given in Table 1. All LEDs are first sequentially sampled (phases #1 to #9), and then, specific wavelengths with higher attenuation or lower output power (740, 760, 780, 910, and 1050 nm) are repeated for signal averaging. As all data collection phases occur within the 10 ms acquisition time, phase repetitions effectively occur simultaneously on the hemodynamic physiological timescale and do not introduce timing uncertainty into the analysis. After each cycle, the microcontroller reads optical data from the AFE, averages multiply sampled wavelength signals, and samples the accelerometer and thermistor before data transmission. For wavelengths measured in multiple phases, averaging maintains the mean signal level while reducing noise. The output data stream contains one value per wavelength for each of the four detectors, an ambient value per detector, the three accelerometer axes, and one temperature value per acquisition cycle.

**Table 1:** LED wavelength assignment for each acquisition phase. Repeat phases are performed at the end of each cycle to improve SNR(√n) and are averaged in the microcontroller, so the output data corresponds to the nine unique LEDs.

Phase #	1	2	3	4	5	6	7	8	9	10	11	12	13	14	15	16
λ (nm)	740	760	780	810	850	890	910	940	1050	740	760	780	910	910	1050	1050

### 2.3 Device Performance Characterization

GS-FlexNIRS performance was characterized in terms of noise performance, dynamic range, signal-to-noise ratio (SNR), temporal stability, and gain linearity. Gain linearity was evaluated by measuring the relationship between detector transimpedance amplifier (TIA) gain settings and the detected light intensity across the range used for long-separation measurements (TIA feedback resistors from 100  kΩ to 1  MΩ). In addition, a thermal safety test was conducted while the device was worn on the forehead.

### 2.4 Wavelength-Weighted Fitting (WWF) Algorithm

To enable quantitative chromophore estimation with spectrally broad LEDs and PDs, the multidistance implementation of a wavelength-weighted fitting (WWF) algorithm for LED-based multiwavelength NIRS systems^35^ was used. Unlike conventional discrete-wavelength approaches that approximate each LED by a single peak or centroid wavelength,^21^^,^^36^^,^^37^ measured LED emission spectra, wavelength-dependent photodiode responsivity, and individually measured source–detector separations are explicitly included to model the detected intensity. The effective attenuation coefficient for each LED channel is estimated from the measured data and LED-specific source–detector separations,^38^ and chromophore concentrations are determined by minimizing the difference between the modeled^39^ and measured effective attenuation coefficients across the nine LED channels. In addition, the WWF algorithm forward model requires either measured or assumed spectrally resolved reduced scattering coefficients. For liquid phantom experiments, these were obtained from simultaneously measured frequency-domain NIRS (FD-NIRS) data. For human measurements, the FD-NIRS measurement was not performed, so literature-based values were assumed instead.^40^ The long source–detector separations (nominally 2.5, 3.0, and 3.5 cm) were used for multidistance fitting. A more detailed description of the algorithm is provided in Sec. S3 of the Supplementary Material.

### 2.5 Liquid Phantom Experiments

Liquid phantom experiments were conducted to validate the ability of the GS-FlexNIRS to quantify hemoglobin and water fraction changes under controlled conditions. Two complementary phantom experiments were performed: (1) validation of hemoglobin saturation changes using a blood-yeast phantom during yeast-induced deoxygenation, and (2) validation of water fraction recovery using blood-glycerol phantoms with varying water content. An FD-NIRS system (MetaOx, ISS Inc., Champaign, Illinois, United States) with eight wavelengths between 670 and 830 nm^41^ was used as a reference in both experiments. Measurements were performed in a custom 3D-printed container (12.8×12.8×11  cm) with optical window cutouts sealed with transparent film (Suresite 123, Medline, Northfield, Illinois, United States) to ensure minimally disrupted optical measurements, and a magnetic stirrer was used to maintain phantom homogeneity.^35^ The temperature of the phantoms was allowed to equilibrate with room temperature (∼19°C) and monitored continuously during the measurements, with minimal variation noted (±0.1°C). The GS-FlexNIRS and multidistance FD-NIRS probes were mounted on opposite windows, facing each other, after verifying minimal optical crosstalk between the two systems.

#### 2.5.1 Liquid phantom 1: hemoglobin validation

A blood-yeast liquid phantom was used to validate GS-FlexNIRS measurements of HbO and HbR during progressive deoxygenation. The phantom consisted of deionized water (DIW), phosphate-buffered saline (PBS), 20% Intralipid solution, bovine blood (#7200806, Lampire Biological Labs, Pipersville, Pennsylvania, United States), and baker’s yeast (YSCII, Sigma-Aldrich, St. Louis, Missouri, United States), with proportions provided in Table 2, adapted from Li et al.^42^ In addition to continuous temperature monitoring, the pH of the phantom was also monitored during the deoxygenation.

**Table 2:** Composition of the blood-yeast phantom. Yeast was added after baseline measurements to initiate deoxygenation.

Phantom component	Amount
PBS + DIW (ml)	750+750 (Total=1500)
20% Intralipid (ml)	80
Bovine blood (ml)	12
Yeast (g)	12

Baseline optical properties were estimated using a calibration-free, translational FD-NIRS configuration (${\mathrm{SDS}}_{\min}=1.97\;\mathrm{cm}$, ${\mathrm{SDS}}_{\max}=2.77\;\mathrm{cm}$, ΔSDS=0.2  cm).^43^^,^^44^ These optical property estimates were used to calibrate the multidistance FD-NIRS measurements, which included four fixed source–detector separations (1.5, 2.0, 2.5, and 3.0 cm) and provided absolute concentration measurements during deoxygenation. Data from the GS-FlexNIRS and FD-NIRS were acquired simultaneously and continuously throughout the deoxygenation process. Yeast was added after 5 min of baseline acquisition, and measurements continued for ∼25  min in the sealed container. FD-NIRS hemoglobin concentrations were estimated assuming a water volume fraction of 100% as the FD-NIRS system does not include wavelengths sensitive to water absorption and the contents of the phantom were known. Reduced scattering values measured by FD-NIRS were parameterized using the empirical power-law relationship,^45^ $\left(\mu_{s}^{\prime }\left(\lambda \right)=a{\left(\frac{\lambda }{\lambda_{0}}\right)}^{-b}\right)$. Two approaches were used to apply the measured scattering values to the GS-FlexNIRS data: (1) fixed scattering measured during the baseline period and (2) time-varying scattering measured throughout the experiment. These scattering values were used as inputs to the WWF algorithm to fit the concentration changes measured by the GS-FlexNIRS.

#### 2.5.2 Liquid phantom 2: water fraction validation

Blood-glycerol phantoms were used to assess the ability of the GS-FlexNIRS to detect changes in water fraction in the presence of hemoglobin absorption. To modify the liquid phantom water fraction, glycerol was selected to replace the water as it is miscible and nonreactive with water, has a distinct near-infrared (NIR) absorption spectrum, and is established for use in tunable liquid optical phantoms.^46^^,^^47^ Four distinct liquid phantoms were prepared with a range of water fractions between ∼60% to 100% (Table 3). Total phantom volume was maintained across formulations to minimize boundary effect differences on the measurements. The PBS and blood volumes were also held constant to preserve a consistent buffer, ionic, and hemoglobin background. The absorption spectrum of glycerol (99%, PTI Process Chemicals, Ringwood, Illinois, United States) was measured prior to the experiments and incorporated into the WWF algorithm for water fraction recovery (Fig. S5 in the Supplementary Material). As previously described,^47^ to maintain consistent optical scattering across phantoms, the amount of intralipid was increased in higher glycerol fraction phantoms to address the reduction in scattering caused by the higher index of refraction of glycerol.^48^ Nominal water fraction was calculated as nonglycerol volume divided by total phantom volume. GS-FlexNIRS measurements were compared against the known water/glycerol composition and reference translational FD-NIRS measurements.

**Table 3:** Composition of the four blood-glycerol phantoms.

Phantom component	Phantom 1	Phantom 2	Phantom 3	Phantom 4
PBS (ml)	750	750	750	750
DIW (ml)	755	535	300	10
Glycerol (ml)	0	200	400	600
20% intralipid (ml)	80	104	142	225
Bovine blood (ml)	12	12	12	12

Each phantom was measured once with translational FD-NIRS and three times using the GS-FlexNIRS. FD-NIRS-derived absorption coefficients were used to compute reference hemoglobin concentrations, with the refractive index and absorption contributions of the solvent approximated as a linear combination of the water and glycerol fractions from the recipe.^48^ GS-FlexNIRS data were analyzed using the WWF algorithm, with the refractive index and FD-NIRS-derived reduced scattering coefficients provided as inputs.

### 2.6 Healthy Human Measurements

A pilot study was conducted in six healthy participants to evaluate the ability of the GS-FlexNIRS to quantify changes in hemoglobin concentration and water fraction *in vivo*. The sample size was selected to demonstrate *in vivo* feasibility rather than to test a physiological hypothesis. The study was approved by the Mass General Brigham Institutional Review Board (IRB, Protocol #: 2020P002463) and conducted in accordance with the Ethical Principles and Guidelines for the Protection of Human Subjects (Belmont Report). Healthy adult volunteers were recruited from the local community through the Mass General Brigham Rally research recruitment website. Exclusion criteria included a BMI above $30\;\mathrm{kg}/m^{2}$, neurological conditions or neuroactive medications, cardiovascular or peripheral vascular disease, and other conditions affecting the ability to complete the study procedures safely. Written informed consent was obtained from all participants prior to participation. Five of the six enrolled participants were included in the final analysis; one was excluded for nonadherence to the breathing protocol, confirmed by the respiratory-belt recording. The analyzed group comprised four female and one male participant, aged 19 to 25 years. Measurements were conducted in a laboratory with participants seated on a reclining chair and the overhead room lights switched off. The sensor was positioned on the lateral forehead, at least 3 cm above the eyebrow to avoid the frontal sinuses and the midsagittal line and to maximize sensitivity to the frontal cortex hemodynamics.^49^ The exact lateral position was adjusted per participant to the flattest available area, minimizing sensor bending. The device was secured with an elastic headband that also holds the battery (Fig. S1 in the Supplementary Material). During sensor placements, signal quality was verified in real time. LED drive currents and detector gains were adjusted to keep detected intensities within the linear range of the detectors, and the sensor was repositioned until the linearity of $\ln(\rho^{2}I(\lambda ))$ versus ρ across the three long separations reached $R^{2}>0.99$ (Sec. S3 of the Supplementary Material). The effect of motion was minimal for these seated tasks, so no motion correction was applied, and no data were excluded on this basis.

Participants performed two breathing maneuvers, repeated breath-holding and paced hyperventilation, to induce reproducible changes in cerebral oxygenation. The breath-holding task consisted of a 1-min baseline followed by four repetitions of a 20-s breath hold and 40 s of normal breathing. Each breath hold was preceded by a 5-s exhalation to minimize residual lung volume. The hyperventilation task began with a 1-min baseline, followed by 1-min of paced hyperventilation at 40 breaths per min guided by an auditory cue, and concluded with a 2-min recovery period of normal breathing. This breathing rate was selected to induce a controlled respiratory challenge while avoiding the pronounced cardiovascular response observed at higher rates in previous studies.^50^^,^^51^

Arterial blood pressure was measured continuously using a noninvasive Finapres system (NOVA, Finapres Medical Systems, Enschede, Netherlands). Additional physiological signals, including electrocardiography, pulse oximetry, and respiration, were acquired using BIOPAC modules (BIOPAC Systems Inc., Goleta, California, United States) and digitized using a synchronized, multichannel analog-to-digital converter (PowerLab 16/30, ML880, ADInstruments, Sydney, Australia). Task timing and participant cues were controlled using PsychoPy (Open Science Tools Ltd., Nottingham, United Kingdom).

GS-FlexNIRS data were post-processed using custom scripts developed in MATLAB. Long SDS intensity data (nominally 2.5, 3.0, and 3.5 cm) were corrected for ambient light and filtered using a fourth order Butterworth lowpass filter with a cutoff frequency of 0.5 Hz to remove cardiac pulsatile oscillations before applying the multidistance WWF algorithm. Reduced scattering coefficients were estimated using power-law parameters from the literature^40^ ($\mu_{s}^{\prime }=8.94(\lambda /750{)}^{-1.10}$).

WWF-estimated water, HbO, HbR, and HbT concentration time series were downsampled to 1 Hz for the correlation analysis. Pearson correlation coefficients (r) were calculated between changes in water fraction ($\Delta H_{2}O$) and changes in each hemoglobin metric, with two-sided p-values testing the null hypothesis of zero correlation, and Pearson’s r was reported as the effect size.^52^ To compare maneuvers, participant-specific correlation coefficients were transformed using the Fisher z-transformation^53^ and analyzed using paired exact two-sided Wilcoxon signed-rank tests,^54^^,^^55^ with matched-pairs rank-biserial correlations ($r_{\mathrm{rb}}$) reported as effect sizes.^56^ With n=5, the exact test has a minimum attainable p-value of 0.0625, so these comparisons are reported descriptively rather than as significance tests.

## 3 Results

### 3.1 LED Emission Spectra and Wavelength Selection

The emission spectra of the LEDs were measured using a near-infrared spectrometer (STS-NIR-L-25-400-SMA, Ocean Optics, Orlando, Florida, United States), accounting for the spectrometer’s spectral response function, to obtain accurate spectral profiles for use in the WWF algorithm (Fig. 2). The measured spectra showed good agreement with the nominal peak wavelengths specified by the LED manufacturer. In addition to LED emission, the wavelength-dependent photodiode responsivity from the manufacturer’s datasheet was incorporated into the inverse model to compute the effective detected spectra. The detected spectrum for each LED therefore reflects the combined effects of LED emission, tissue absorption, and photodiode sensitivity.

**Fig. 2:**
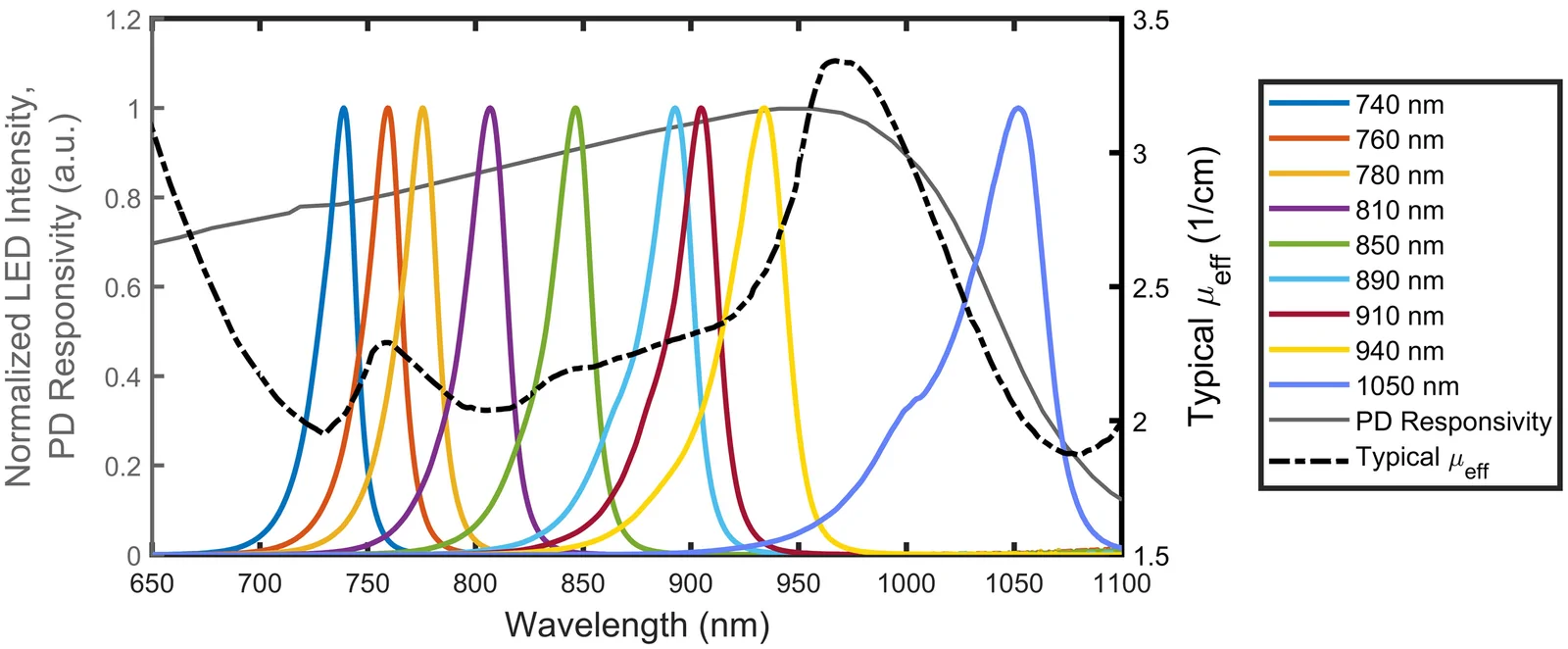
Left axis: Normalized LED emission spectra measured with a near-infrared spectrometer (STS-NIR-L-25-400-SMA, Ocean Optics) after correction for the spectrometer’s spectral response function, and the normalized spectral responsivity of the photodiode (VEMD5080X01, Vishay Intertechnology, Inc.) from the datasheet. Right axis: Typical effective attenuation coefficient spectrum in human tissue, assuming HbO=51.5  μM, HbR=29.8  μM, water=80%, and scattering $\mu_{s}^{\prime }=8.94(\lambda /750{)}^{-1.10}$.

As can be seen in Fig. 2, we have avoided using wavelengths in the strong absorption feature of water between 970 and 980 nm. Although wavelengths near the water absorption peak (970 to 980 nm) provide maximal intrinsic sensitivity to water, the combination of strong absorption and broad LED spectral bandwidth causes severe signal attenuation and spectral distortion. Under typical tissue conditions, most photons near the water peak are absorbed, so the detected signal is dominated by the spectral tails rather than the intended wavelength. This spectral deformation substantially reduces effective signal strength and increases sensitivity to modeling errors (Fig. S3 in the Supplementary Material). LEDs at 910, 940, and 1050 nm were therefore selected as a compromise: they preserve meaningful water sensitivity while avoiding the distortion observed closer to the absorption peak. Combined with the spectrum-conscious WWF framework, this selection enables reliable water fraction estimation in an LED-based wearable NIRS system.

### 3.2 Instrument Performance Characterization

Across all wavelengths and detector channels, the noise-equivalent power (NEP) ranged from 131.9 to 158.0  fW/√Hz. The system supported maximum detectable optical intensities of 14 to 16 nW, corresponding to a dynamic range of ∼100  dB [Fig. S2(a) in the Supplementary Material]. Across the TIA gain range used for long-separation measurements, measured light intensity scaled linearly with nominal TIA gain ($R^{2}>0.99997$). Under liquid phantom conditions (ink, intralipid, and water; Table S1 in the Supplementary Material) at a 3.5 cm source–detector separation, unfiltered SNR values ranged from ∼67 to 80 dB, whereas the short-separation channels achieved values as high as 90 dB [Fig. S2(b) in the Supplementary Material]. All measured SNR exceeded the 60-dB SNR benchmark cited by Wyser et al. for continuous-wave functional near-infrared spectroscopy instruments^57^ and are well above the 40-dB range reported for several working systems.^57^^–^^60^ Long-term stability testing showed intensity variations below ±2.5% over periods exceeding 6 h, compatible with overnight recordings. A 1-h wear trial confirmed thermal compliance of the device. The LED array and AFE plateaued at 37.6°C within ∼15  min of device activation, below the limit for prolonged skin contact,^61^ whereas skin adjacent to the device remained at 32.4°C (Fig. S4 in the Supplementary Material).

### 3.3 Hemoglobin Deoxygenation Validation

Time courses of the fit changes in HbO, HbR, HbT, and $H_{2}O$ from both instruments can be seen in Fig. 3. In Fig. 3, t=0 corresponds to ∼6  min 40 s after yeast addition, once the phantom appeared homogeneous and the measured intensities stabilized following the yeast-induced increase in phantom scattering, with changes being assessed relative to the average of the first minute from t=0. Under the fixed-scattering assumption, GS-FlexNIRS estimated an end-deoxygenation 3.93% increase in water fraction, with end-deoxygenation deviations of −0.05  μM in HbO and +0.88  μM in HbR compared with FD-NIRS [Fig. 3(a)]. Though, a gradual reduction in scattering was detected by FD-NIRS over the deoxygenation period (Fig. S6 in the Supplementary Material), and when accounted for, the estimated water fraction increase at end-deoxygenation was 1.36%, with the end-deoxygenation hemoglobin deviations of −0.03  μM in HbO and +1.39  μM in HbR [Fig. 3(b)] as compared with FD-NIRS. The apparent water fraction changes in the fixed-scattering case suggest crosstalk between absorption and scattering, which could be as a result of the observed pH change^62^ through the deoxygenation (7.28→6.48) or oxygenation-dependent changes in blood scattering during deoxygenation.^63^^,^^64^ The root mean square error of the WWF fit was comparable under both scattering conditions, increasing modestly over the course of deoxygenation from 0.0083 to $0.0109\;{\mathrm{cm}}^{-1}$ with fixed scattering and to $0.0114\;{\mathrm{cm}}^{-1}$ with time-varying scattering. Correspondingly, the coefficient of determination decreased slightly from 0.9989 to 0.9966 and 0.9963, respectively.

**Fig. 3:**
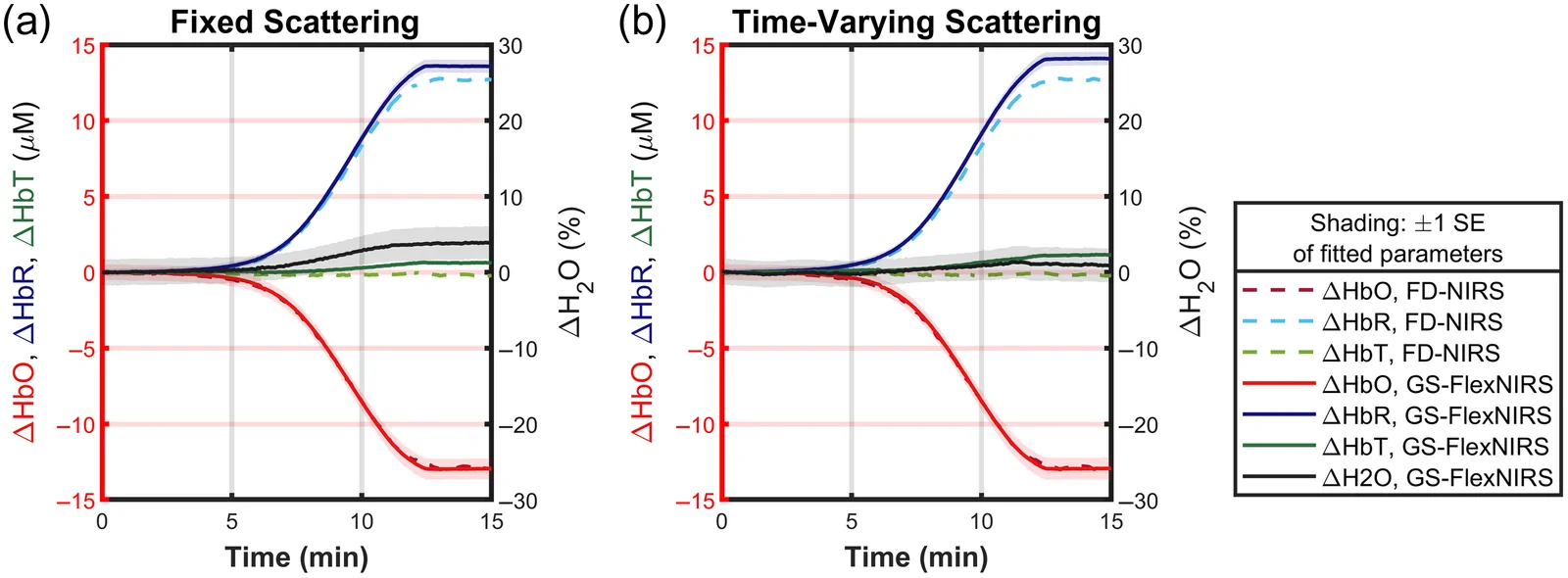
Blood–yeast phantom deoxygenation measured with GS-FlexNIRS and reference FD-NIRS. GS-FlexNIRS results are shown using (a) fixed reduced scattering coefficients and (b) time-varying reduced scattering coefficients measured by FD-NIRS. In both panels, GS-FlexNIRS estimates of ΔHbO, ΔHbT, and ΔHbR (μM) are plotted alongside FD-NIRS reference values, together with the estimated water fraction change ($\Delta H_{2}O$, %). Shaded bands indicate the standard error of the GS-FlexNIRS parameter estimates obtained from the nonlinear WWF fit.

### 3.4 Water Fraction Phantom Validation

The GS-FlexNIRS tracked the reductions in water fraction across phantoms with decreasing water content, with recovered $\Delta H_{2}O$ linearly related to the expected values ($R^{2}=0.966$) and a fitted slope of 0.790 [Fig. 4(c)]. Observed differences in hemoglobin concentration measurements made relative to phantom 1 were small for both GS-FlexNIRS and FD-NIRS measurements [Figs. 4(a) and 4(b)], indicating that variations in solvent composition (water versus glycerol) did not introduce significant hemoglobin crosstalk, with GS-FlexNIRS deviations comparable in magnitude to those of FD-NIRS.

**Fig. 4:**
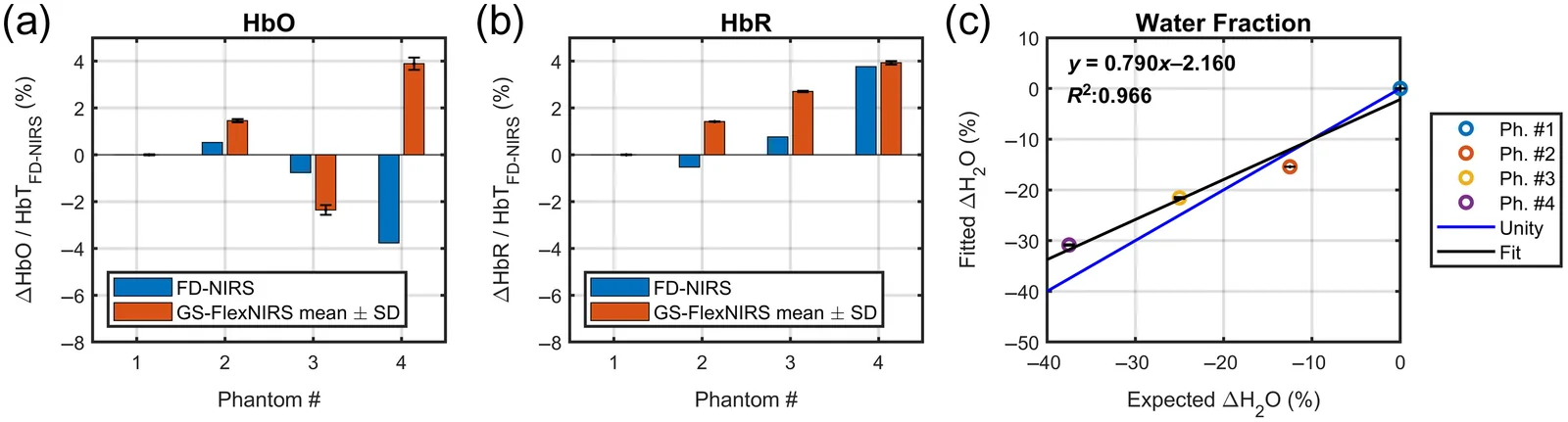
Results from the four blood-glycerol phantoms. Changes in FD-NIRS–HbT–normalized (a) oxyhemoglobin (ΔHbO/HbT) and (b) deoxyhemoglobin (ΔHbR/HbT) relative to phantom 1. (c) Water fraction changes estimated by GS-FlexNIRS, plotted against expected values based on phantom composition; the blue line denotes unity, and the black line the fitted relationship. Each data point corresponds to one phantom formulation (n=4), measured three times. Error bars represent ±1 standard deviation across the three repeated measurements for GS-FlexNIRS. In panel (c), the error bars are smaller than the plotted markers.

### 3.5 Validation on Healthy Human Participants

*In vivo*, hemoglobin concentration changes captured by the GS-FlexNIRS (Fig. 5) were consistent with those reported in our previous study.^50^ During breath-holding, ΔHbO and ΔHbT increased while ΔHbR decreased, consistent with a compensatory increase in cerebral blood flow,^65^ leading to increased cerebral blood volume above baseline. During hyperventilation, both ΔHbO and ΔHbT decreased. The second phase of the biphasic hemodynamic response reported previously was absent here likely because of the lower hyperventilation rate (40 versus 70 breaths per minute). In both breathing maneuvers, $\Delta H_{2}O$ was positively correlated with ΔHbO in every participant (r=0.44 to 0.97). Correlation with ΔHbT was also positive but more variable (r=0.20 to 0.95), and correlations with ΔHbR were inconsistent (r=−0.78 to +0.51) (Fig. S7 in the Supplementary Material).

**Fig. 5:**
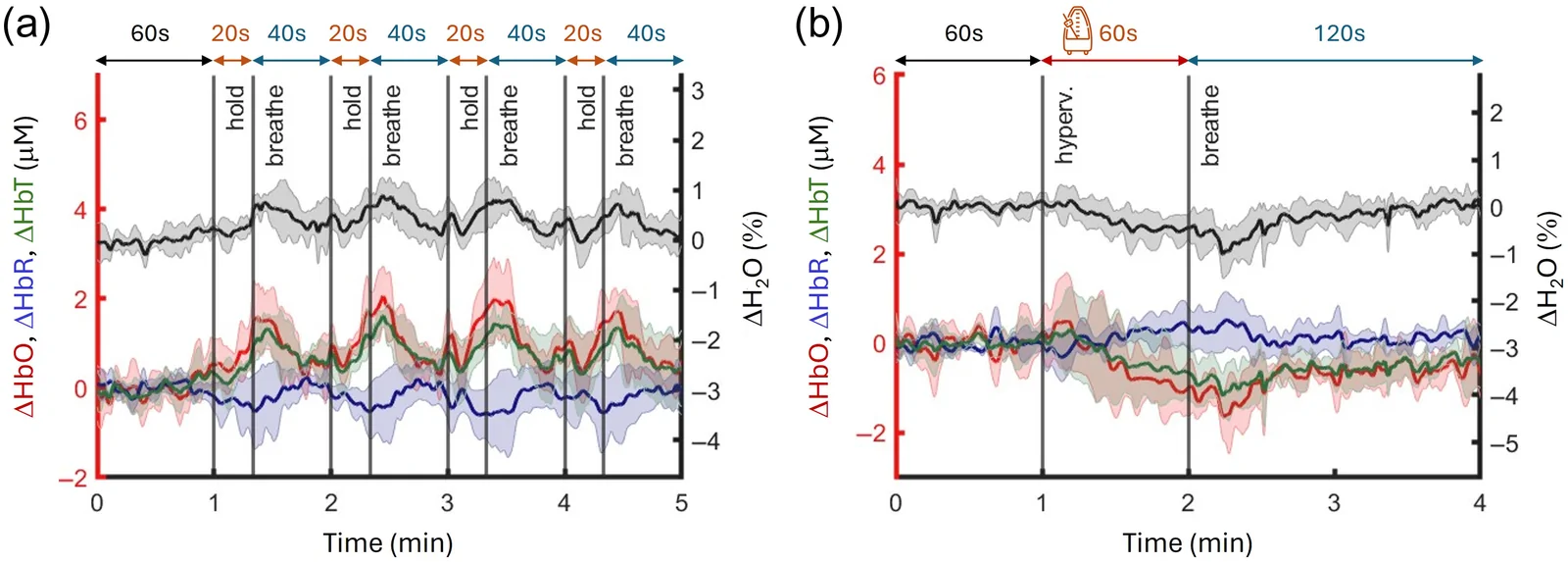
Hemoglobin and water fraction changes during (a) repeated breath-holding and (b) hyperventilation in five participants. Traces are derived from the multidistance fit across the three long source–detector separations, measured on the lateral forehead overlying frontal cortex. ΔHbO, ΔHbR, and ΔHbT are in μM; $\Delta H_{2}O$ is in percent. Bold traces show group averages (n=5); shaded regions indicate standard deviation across participants. Period labels above the plots are color coded: baseline in black, stimulus period in orange, and rest in dark teal.

Correlations tended to be higher during breath-holding than during hyperventilation for the HbO–water and HbT–water comparisons, with matched-pairs rank-biserial effect sizes of 1.000 and 0.600, respectively; the HbR–water effect size was near zero (0.067). As noted in Sec. 2.6, the minimum attainable exact two-sided p at n=5 is 0.0625; these comparisons are therefore reported descriptively (Table 4).

**Table 4:** Comparison of correlation coefficients between breath-holding and hyperventilation. Participant-specific Pearson correlation coefficients between each hemoglobin metric and $\Delta H_{2}O$ were Fisher r-to-z transformed and compared using paired exact two-sided Wilcoxon signed-rank tests.

Correlation pair	$W^{+}$	$n_{\text{pair}}$	Exact p	$r_{\mathrm{rb}}$
ΔHbO and $\Delta H_{2}O$	15.0	5	0.0625	1.000
ΔHbR and $\Delta H_{2}O$	8.0	5	1.0000	0.067
ΔHbT and $\Delta H_{2}O$	12.0	5	0.3125	0.600

Paired differences were calculated as breath-holding minus hyperventilation. $W^{+}$ is the sum of ranks corresponding to positive paired differences; $n_{\text{pair}}$ is the number of nonzero paired differences; p is the exact two-sided p-value; $r_{\mathrm{rb}}$ is the matched-pairs rank-biserial correlation (effect size). Positive values of $r_{\mathrm{rb}}$ correspond to larger participant-specific correlations during breath-holding. With n=5, the minimum attainable exact two-sided p-value is 0.0625.

## 4 Discussion

We describe the GS-FlexNIRS, a low-cost, wearable NIRS system capable of simultaneously tracking hemoglobin concentration changes and tissue water fraction at a sampling rate of 100 Hz. To our knowledge, this is the first wearable NIRS device to achieve concurrent hemoglobin and water fraction monitoring at this temporal resolution and signal-to-noise performance, extending prior demonstrations in broadband and laser-based CW-NIRS systems, which were primarily designed for benchtop or controlled settings rather than wearable applications.^25^^–^^27^ The flexible PCB form factor and wireless BLE operation of the GS-FlexNIRS are compatible with deployment in naturalistic settings for longitudinal measurements where cost and wearability are primary constraints. Furthermore, as was the case with the first-generation FlexNIRS device, the per unit cost of the GS-FlexNIRS device is ∼$350 for small (10) batch production. A detailed cost breakdown is provided in Sec. S2 of the Supplementary Material. This price point supports large-scale studies, though comparison with commercial systems should account for coverage: GS-FlexNIRS provides a single frontal measurement location rather than multiregion or whole-head coverage.

The GS-FlexNIRS achieves SNR, dynamic range, and long-term stability comparable to previous FlexNIRS generations.^33^^,^^35^^,^^50^ Its noise-equivalent power of 131.9 to 158.0  fW/√Hz indicates lower sensitivity than the original FlexNIRS (57.9 and 66.4  fW/√Hz)^33^ and ninjaNIRS (36.4  fW/√Hz),^66^ but substantially higher sensitivity than MOBI (7.3 to 8.9  pW/√Hz),^67^ among compact, fiberless wearable systems using silicon-photodiode detection where bandwidth-normalized NEP has been reported. Reported NEP values also depend on acquisition timing, averaging, and estimation procedures, which differ across these systems.^33^^,^^66^^,^^67^

Extending NIRS measurements to include tissue water fraction using LEDs presents unique challenges. Water exhibits strong and sharply varying absorption features in the near-infrared wavelength range, particularly near 980 nm, which interact unfavorably with the broad emission bandwidth of LEDs (Fig. S3 in the Supplementary Material). By targeting wavelengths at 910, 940, and 1050 nm and applying the WWF algorithm, the GS-FlexNIRS preserves sensitivity to water fraction. The multidistance implementation provides further advantages, namely, a built-in linearity quality metric ($R^{2}$ of $\ln(\rho^{2}I(\lambda ))$ versus ρ) and a relative insensitivity to source power drift.^33^

Device accuracy was first evaluated in controlled liquid phantom experiments. In the blood-yeast phantom, GS-FlexNIRS tracked progressive hemoglobin deoxygenation in agreement with the reference multidistance FD-NIRS measurements. The small apparent increase in water fraction observed under the fixed-scattering assumption was attributed to absorption-scattering crosstalk, and incorporating time-varying scattering measured by FD-NIRS reduced this deviation. Although the drift in the accuracy of the instrument is a concern, similar scattering changes have been reported in measurements of blood-yeast phantoms^68^ and are likely driven by yeast aggregation and the large oxygenation swing.^63^ Neither of these effects is likely to be present in a typical measurement of naturalistic sleep, and as a result, are less concerning overall.

In the blood-glycerol phantoms, water fraction sensitivity was assessed by systematically varying solvent composition over a super-physiologic range of 37.5%. Recovered water fraction reductions were linearly related to the expected values ($R^{2}=0.966$) with a fitted slope of 0.790, indicating that the imposed changes were tracked in proportion but underestimated in magnitude.

In healthy participants, hemoglobin responses during controlled respiratory manipulations were consistent with prior studies, including increases in ΔHbO and ΔHbT during breath-holding and decreases during hyperventilation.^33^^,^^50^ The relationship between water and hemoglobin changes was stronger during breath-holding than during hyperventilation, though the difference cannot be tested at this sample size. GS-FlexNIRS provides spectral sensitivity to water; however, the estimated water fraction reflects combined water contributions rather than individual fluid compartments. The positive correlations with HbO and HbT suggest a vascular contribution but cannot distinguish whether it is from changes in CSF or other tissue water compartments. Perturbations that dissociate blood volume from tissue water content, together with independent reference measurements, would be needed to disentangle these contributions and to develop and validate future compartment-aware algorithms.

The study has several limitations. In the glycerol-intralipid phantoms, the difference in solvent refractive index required compensation of the scattering coefficient. Future work may benefit from alternative phantom formulations with reduced spectral overlap, such as heavy water^69^ or gelatin.^70^ In human measurements, reduced scattering coefficients were not measured directly but assumed from literature values, which may introduce systematic uncertainty. Scattering changes in the adult brain during these tasks are expected to be small, however, and fixed-scattering assumptions are common in CW-NIRS analysis. In addition, although short-separation measurements were collected in these experiments, short-separation regression or other systemic physiology regression was not performed on the collected data due to the systemic nature of the stimuli, though including regression is a natural extension of the current pipeline in future *in vivo* studies.^21^^,^^71^^,^^72^ Although the device is well suited for at-home monitoring in theory, all measurements were performed in a laboratory setting, so the suitability of the system for naturalistic, at-home monitoring remains to be demonstrated.

## 5 Conclusion

Taken together, these results show that tissue water fraction monitoring is feasible using a low-cost, LED-based wearable NIRS system when combined with appropriate wavelength selection and spectrum-conscious analysis. In phantom experiments, the GS-FlexNIRS resolved water fraction changes independently of hemoglobin, with recovered values consistent with reference FD-NIRS measurements. In human participants, the system captured hemoglobin dynamics consistent with expected trends during respiratory challenges. These findings support the viability of wearable water fraction monitoring and motivate longitudinal, naturalistic studies of brain fluid dynamics, with potential applications in sleep, hydration, edema, and glymphatic system function.

## Supplementary Material

10.1117/1.JBO.31.10.106006.s01

## Data Availability

The data that support the findings of this study are available from the corresponding author upon reasonable request. The analysis code used in this study is available from the corresponding author upon reasonable request.
