# Supplementary material for: Development and validation of a low-cost, wearable near-infrared spectroscopy system for quantifying tissue water fraction in the human brain
Source: J Biomed Opt. 2026 Oct 28;31(10):106006. doi: 10.1117/1.JBO.31.10.106006 (PMC13715939; doi:10.1117/1.JBO.31.10.106006)
Supplement: Supplementary file 1 [file JBO_031_106006_SD001.pdf]

Appendix A: Supplemental Material

S1 GS-FlexNIRS placement and attachment

For human measurements, the GS-FlexNIRS device was positioned on the lateral forehead, away from the midsagittal line, and secured with a headband. An integrated pouch in the headband held the rechargeable lithium-polymer (LiPo) battery, which was connected to the device through a magnetic pogo-pin connector.

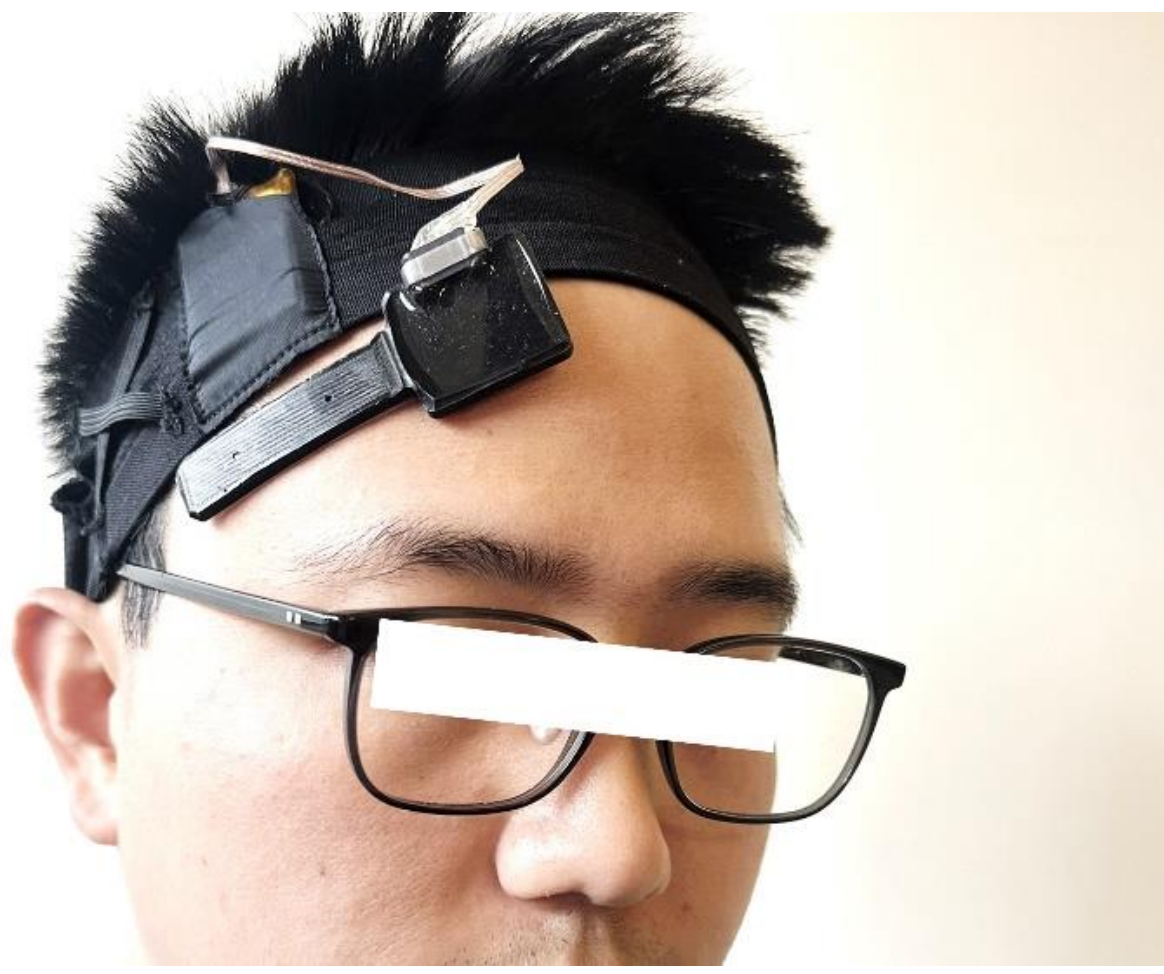

Fig. S1 GS-FlexNIRS placement on the forehead. The optical sensor is positioned on the lateral forehead, at least 3 cm above the eyebrow and away from the midsagittal line, overlying the frontal cortex. The control module, visible near the hairline, contains the electronics and does not require skin contact. The headband is pulled down over the device during use; it has been lifted here to make the device visible.

S2 GS-FlexNIRS device Cost

The total bill-of-materials cost of the GS-FlexNIRS is approximately US$350 per unit for a small batch of 10 units. Flexible printed circuit board fabrication and assembly represent the largest cost component (approximately US$180 per unit) and are expected to decrease with larger production volumes. The nine high-power LEDs constitute the second-largest cost, totaling approximately

US$100, with individual prices ranging from US$6 to US$33 depending on wavelength. The lithium-polymer battery, AFE4460 analog front end, Bluetooth Low Energy microcontroller, and four photodiodes collectively cost approximately US$60. The remaining US$10 covers passive components, connectors, and packaging materials. This estimate excludes the final assembly labor, the tablet, and development costs.

S3 Wavelength-weighted fitting algorithm

The wavelength-weighted fitting (WWF) algorithm incorporates the measured LED emission spectra and wavelength-dependent photodiode responsivity into the forward model. Chromophore concentrations are estimated by fitting the model to measured signals across LED channels and source-detector separations. The original algorithm was described by Otic et al.[1]. The original algorithm includes multi- and single-distance implementations. In this study, we used the multi-distance implementation.

Under the continuous-wave diffusion approximation[2], measured light intensity measurements in the diffuse regime at long source-detector separations ($\rho > 2\ cm$, see Liu et al.[3]) exhibit the approximate relationship between $\ln[\rho^2 I(\rho, \lambda)]$ vs. ρ [3,4]:

$$\ln[\rho^2 I(\rho,\lambda)] \approx -\mu_{eff}(\lambda)\rho + \beta_0(\lambda). \qquad (s1)$$

Here, ρ is the source-detector separation, λ is wavelength, I(ρ,λ) is the detected intensity, $\mu_{eff}(\lambda)$ is the wavelength-dependent effective attenuation coefficient estimated as the negative of the fitted slope, and $\beta_0(\lambda)$ is the fitted intercept. The effective attenuation coefficient is related to the absorption and reduced scattering coefficients by[5]

$$\mu_{eff}(\lambda) = \sqrt{3\mu_a(\lambda)\big(\mu_a(\lambda) + \mu_s'(\lambda)\big)}, \qquad (s2)$$

where μ_a is the absorption coefficient and μ_s' is the reduced scattering coefficient. However, for

an LED with a broad emission spectrum, the estimated μ_{eff} is a weighted term across the distribution of the LED emission spectrum.

The modeled detected intensity was calculated by combining the LED emission spectrum, $I_0(\lambda)$, the photodiode responsivity, $R(\lambda)$, and the attenuation of the light by the tissue, $A(\rho, \lambda)$. We measured the $I_0(\lambda)$ spectrum for each LED and obtained $R(\lambda)$ from the manufacturer's datasheet. For a given set of chromophore concentrations and its corresponding optical properties, $A(\rho, \lambda)$ was calculated using the semi-infinite solution to the light-diffusion equation[5],

$$A(\rho,\lambda) = \frac{3(\mu_a(\lambda) + \mu_s'(\lambda))}{4\pi}\left(\frac{\exp\left(-\sqrt{3\mu_a(\lambda)(\mu_a(\lambda) + \mu_s'(\lambda))}r_1(\rho,\lambda,\mu_s'(\lambda))\right)}{r_1(\rho,\lambda,\mu_s'(\lambda))} - \frac{\exp\left(-\sqrt{3\mu_a(\lambda)(\mu_a(\lambda) + \mu_s'(\lambda))}r_2(\rho,\lambda,\mu_s'(\lambda))\right)}{r_2(\rho,\lambda,\mu_s'(\lambda))}\right). \quad (s3)$$

The reduced scattering coefficient was modeled using the power law[6] $\mu_s'(\lambda) = a\left(\frac{\lambda}{\lambda_0}\right)^b$. The absorption coefficient was modeled as

$$\mu_a(\lambda) = \Sigma\varepsilon_i(\lambda) * c_i + \Sigma\mu_{a,j}(\lambda) * f_j \quad (s4)$$

where for chromophore i, $\varepsilon_{a,i}$ is the extinction coefficient spectrum and c_i is the concentration; for solvent j, $\mu_{a,j}$ is the absorption coefficient spectrum and f_j is the volume fraction. The r terms are distance terms which depend on the source-detector separation (ρ), the tissue reduced scattering coefficient, and the tissue refractive index, as defined in the semi-infinite diffusion model of Farrell et al.[5] and were unchanged in this implementation.

For a set of tissue chromophore concentrations, the detected intensity ($\hat{I}(\rho)$) was modeled for each pair of LEDs and photodetectors:

$$\hat{I}(\rho) = \int_{\lambda_{min}}^{\lambda_{max}} I_0(\lambda)R(\lambda)A(\rho,\lambda)d\lambda \quad (s5)$$

The modeled effective attenuation coefficient for each LED ($\hat{\mu}_{eff}$) was then calculated from the M long-separation detectors:

$$\hat{\mu}_{eff} = -slope_{\rho}\left\{\ln\left(\rho_m^2 \hat{I}(\rho_m)\right)\right\}_{m=1}^{M}, \quad (s6)$$

Chromophore concentrations and solvent volume fraction were estimated by minimizing the nonlinear least-squares loss between the modeled and measured effective attenuation coefficients across the N LED channels,

$$L = \sum_{n=1}^{N}\left(\hat{\mu}_{eff}(n) - \mu_{eff}(n)\right)^2, \quad (s7)$$

where n is the index for each LED and N is the total number of LEDs.

S4 GS-FlexNIRS app and tablet

Modifications to the probe geometry and the number of LEDs, wavelengths, and detectors required development of a new application for GS-FlexNIRS. The existing Android application was updated with the assistance of Etere Studio LLC and was migrated to Flutter (Google, United States) to enable cross-platform compatibility both with Android and iOS devices.

Updates to the application include reporting the linearity (r^2) of $\ln(\rho^2 I)$ as a function of source-detector separation (ρ) and enabling flexible configuration of the number of LEDs, wavelengths, and detectors through a configuration file. For simplicity, the application displays cerebral hemoglobin oxygenation (SO_2) calculated from two selected wavelengths, while water content and hemoglobin concentration changes are computed during post-processing using WWF.

S5 GS-FlexNIRS signal-to-noise ratio

To evaluate the expected SNR of the GS-FlexNIRS instrument in human participants, a phantom was made with optical properties matching typical cerebral tissue. The phantom consisted of a mixture of ink, water, and glycerol for absorption, and intralipid for scattering. Based on linear combinations of absorption spectra of ink, water, and glycerol, the absorption coefficients for the

traditional NIRS wavelengths (740 to 850 nm) ranged from 0.12–0.15 cm^{-1}. At water-sensitive wavelengths, the estimated absorption coefficients were 0.17 cm^{-1} at 910 nm, 0.25 cm^{-1} at 940 nm, and 0.26 cm^{-1} at 1050 nm. Phantom optical properties were validated using the FD-NIRS device (MetaOx, ISS Inc., United States), with reduced scattering coefficients fitted as $\mu_s' = 7.64 (\lambda/750)^{-1.27}$. Measurements were made in the same liquid phantom container described in Sec. 2.5.1. The formulation of the phantom is given in Table S1, and the results of the SNR measurements can be seen in Fig. S2.

Table S1 Components of the liquid phantom used for realistic SNR measurements (Ink: India ink). The phantom was formulated to achieve absorption and reduced scattering coefficients representative of human tissue.

Phantom component	Volume (mL)	Volume Fraction (%)
Water	1931.4	64.4
Intralipid	211.5	7.0
India Ink (1:40 dilution)	107.1	3.6
Glycerol	750.0	25.0
Total	3000	100

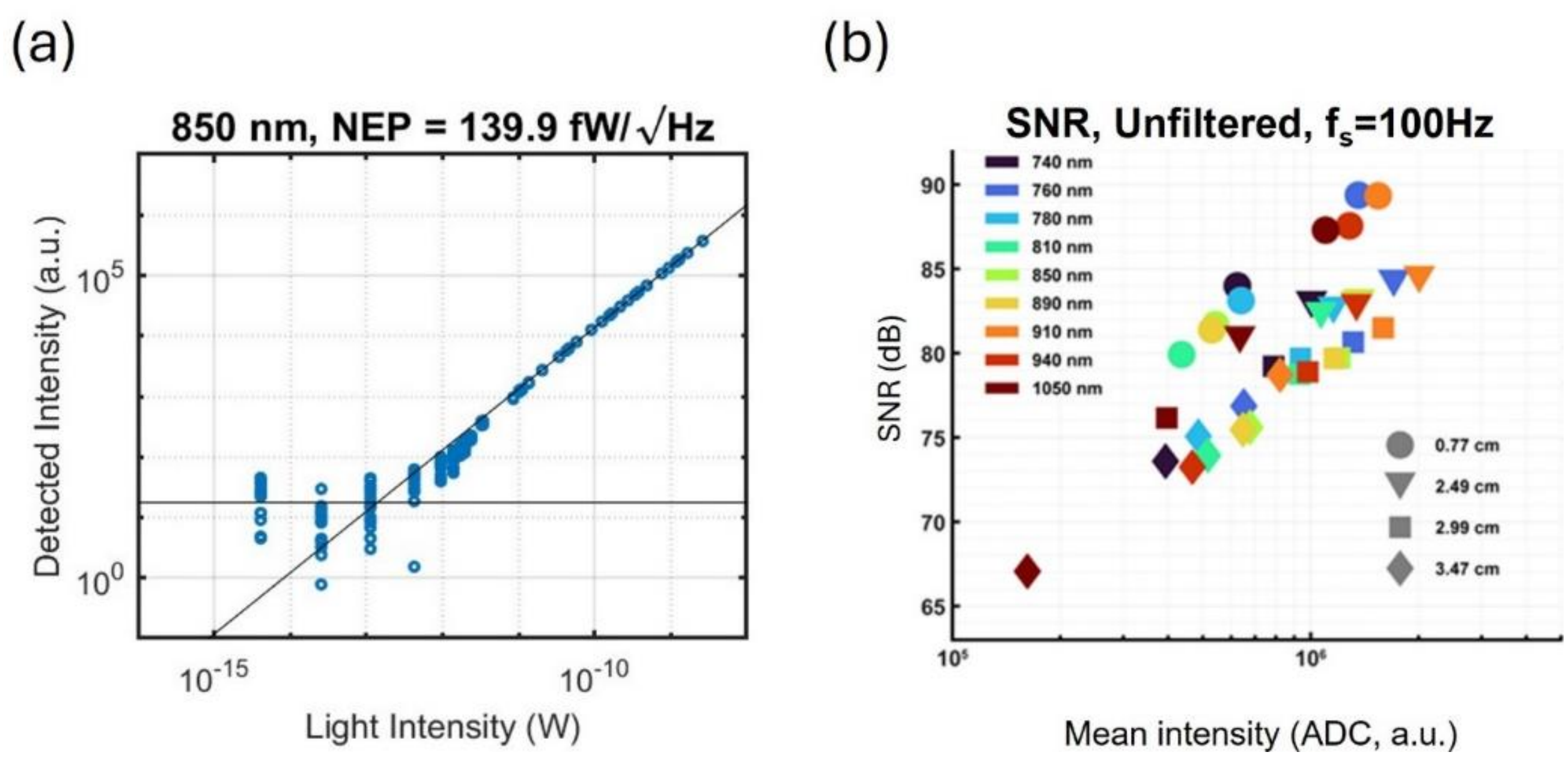

Fig. S2 GS-FlexNIRS characterization: (a) Noise-equivalent power (NEP) measurement example for the 850 nm

LED. (b) Signal-to-noise ratio (SNR) measured on a realistic liquid phantom (ink, intralipid, and water), showing SNR values from 67 to 90 dB, plotted against the mean intensity recorded in the AFE's analog-to-digital converter (ADC) arbitrary unit (a.u.).

S6 Effect of peak water absorption on detected LED spectra

To demonstrate the effect of water's absorption peak between 970 and 980 nm, we have simulated the effective detected spectra of LEDs with nominal wavelengths of 850, 940, 970, and 1050 nm. In Fig. S3(a), we plot the LEDs' emission spectra, the responsivity of the photodiode, and the typical effective attenuation coefficient spectrum for human cerebral tissue, where the intrinsic sensitivity of the 970 nm wavelength LED to the water peak feature is evident given the peak in the attenuation spectrum. However, when simulating the spectra of the detected light (Fig. S3(b)), the light returning from the tissue from the 970 nm LED appears heavily attenuated and distorted, overlapping with the 940 nm LED profile. While the wavelength weighted fitting algorithm would be informed of these effects given the inclusion of the LED spectral shape, photodiode responsivity, and estimated tissue optical properties, we decided against using this wavelength in the system due to this severe spectral distortion.

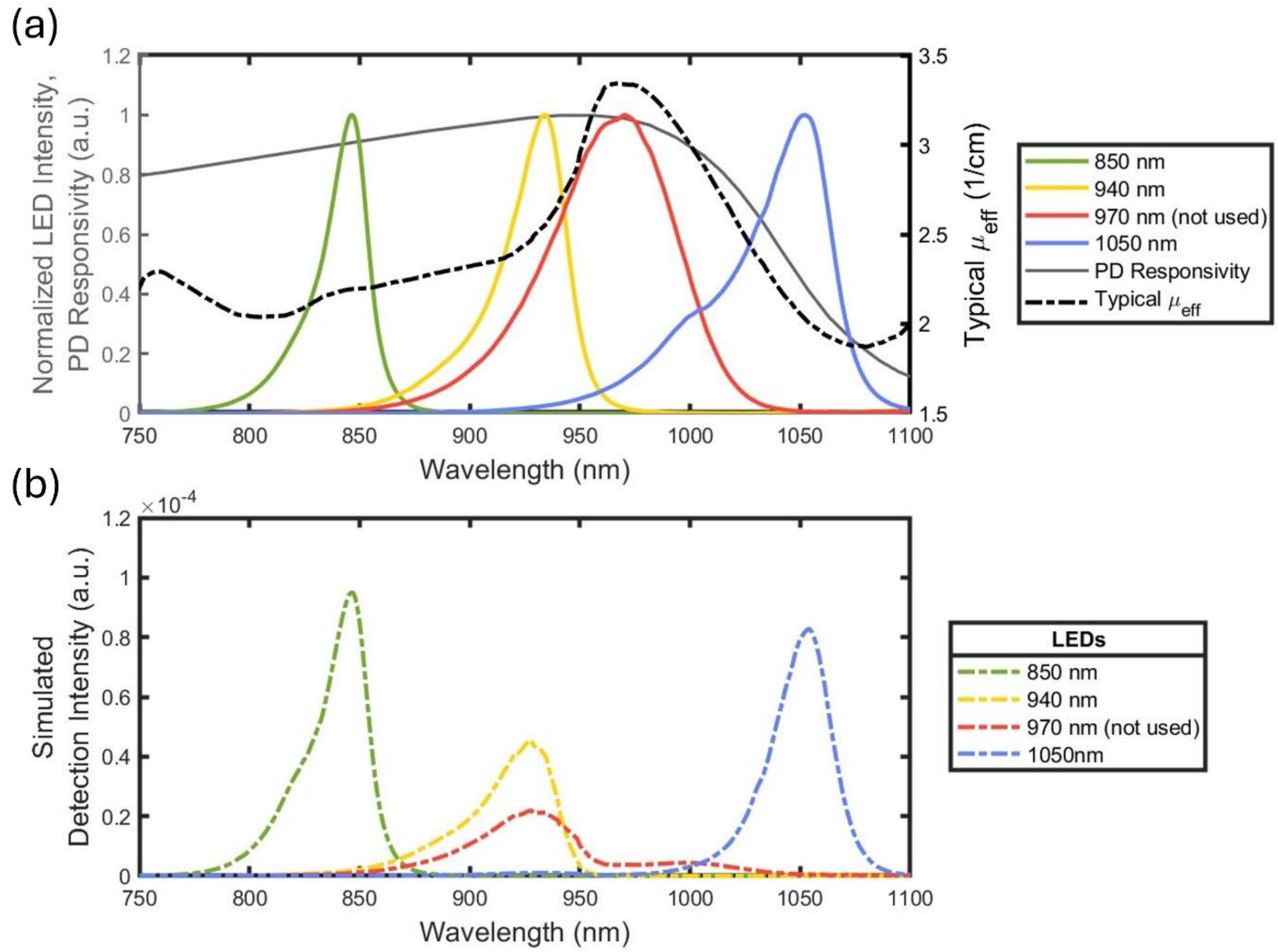

Fig. S3 Simulation of LED spectral distortion when measuring a sample with water, focusing on four LED wavelengths: 850 nm (typical NIRS wavelength), 940 and 1050 nm (left and right shoulder of the water peak; see Fig. S5), and 970 nm (water peak). (a) Left axis: normalized LED emission spectra measured with a near-infrared spectrometer (STS-NIR-L-25-400-SMA, Ocean Optics, United States) after correction for the instrument spectral response function, and the normalized spectral responsivity of the photodiode (VEMD5080X01, Vishay Semiconductors, United States) from the data sheet. Right axis: a typical effective attenuation coefficient spectrum in human tissue, assuming HbO = 51.5 μM, HbR = 29.8 μM, water fraction = 80%, and scattering μ_s' = 8.94 $(\lambda/750)^{-1.10}$. (b) Simulated LED spectral distortion at 3 cm separation, showing minimal distortion at 850 nm, moderate spectral shifts at 940 and 1050 nm, and substantial distortion at 970 nm. The calculated peak shifts were 0.0, -6.3, 2.0, and -42.7 nm, respectively. Due to the severe distortion of 970 nm LED, this wavelength was not included in the GS-FlexNIRS device.

S7 Sensor temperature behavior during operation

To ensure the device presents no thermal hazard to the wearer, we performed a turn-on test and temperature measurement while the device was worn by a volunteer. A four-channel, fiber based thermometer (Reflex-4, NeoOptix Inc., Canada) was used to measure (1) the temperature at the LEDs in contact with the skin, (2) the temperature above the analog front-end on the backside of the GS-FlexNIRS board, opposite from the skin-facing side, (3) the temperature of the skin in a location close to but not underneath the GS-FlexNIRS device, and (4) the ambient temperature during the measurement. Shown in Fig. S4, the LED and analog front-end temperatures rose after device activation and plateaued at 37.6 °C within approximately 15 minutes, while the skin adjacent to the device remained at 32.4 °C. The maximum temperature of the device in skin contact therefore remained below the 42 °C limit for prolonged contact[7].

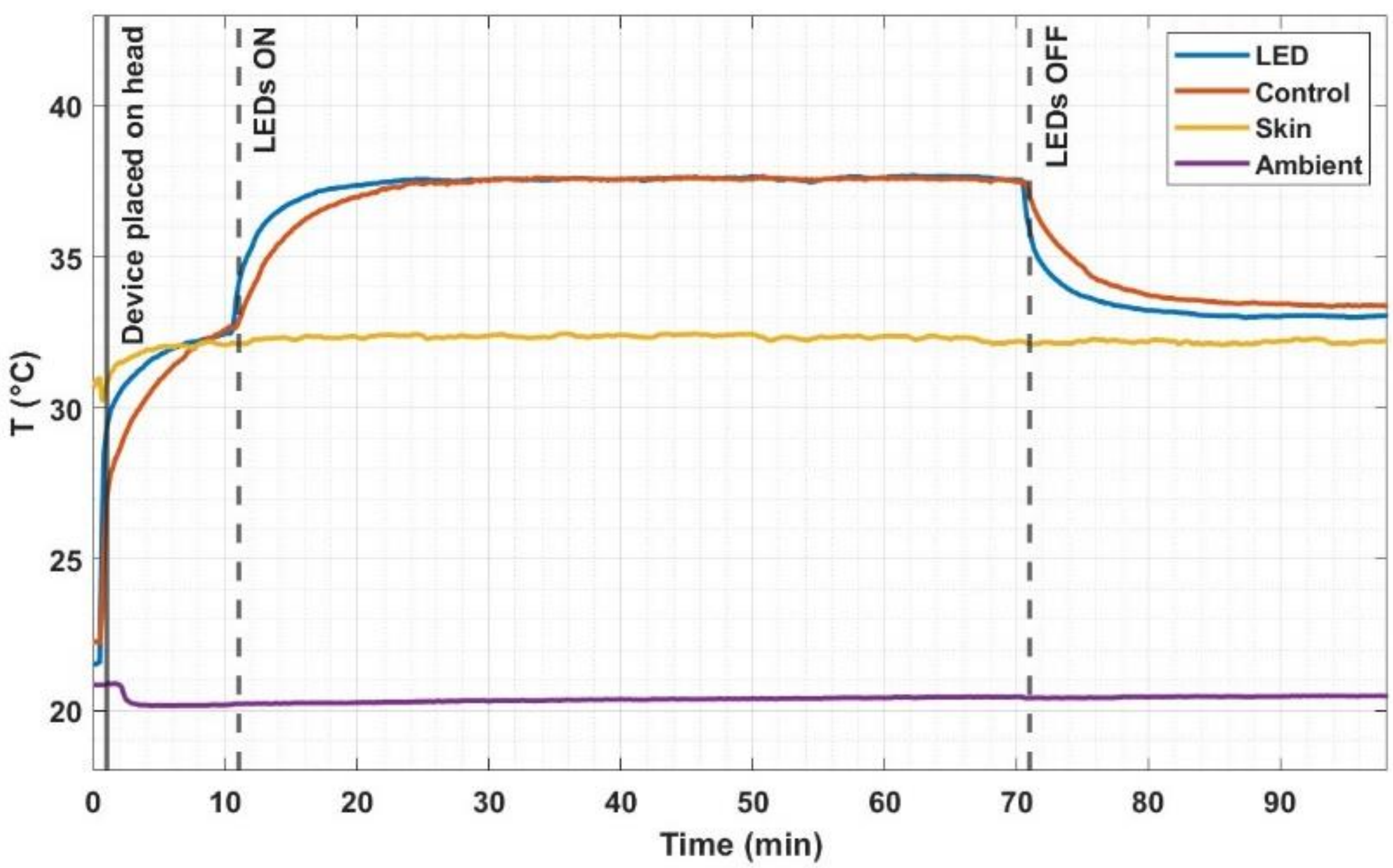

Fig. S4 Temperature measurement over an hour-long period. The temperature of the control analog front-end and the LEDs plateaued at 37.6 °C after 15 minutes of turning on the LEDs while the surrounding skin temperature stayed at 32.4 °C.

S8 Measurement of glycerol and water spectra for use in the wavelength weighted fitting algorithm

To improve the accuracy of the chromophore recovery during the phantom experiments, we prepared samples of water and glycerol transmission absorption measurements. Samples were measured in a 1 cm pathlength cuvette using a tungsten–halogen light source (HL-2000-FHSA, Mikropack GmbH, Germany) and a near-infrared spectrometer (STS-NIR-L-25-400-SMA, Ocean Optics, United States). The measured water absorption spectrum is shown alongside the reference spectrum reported by Kou et al.[8] for validation. These spectra were used in the fitting analysis for the blood-glycerol phantoms presented in Sec. 3.4.

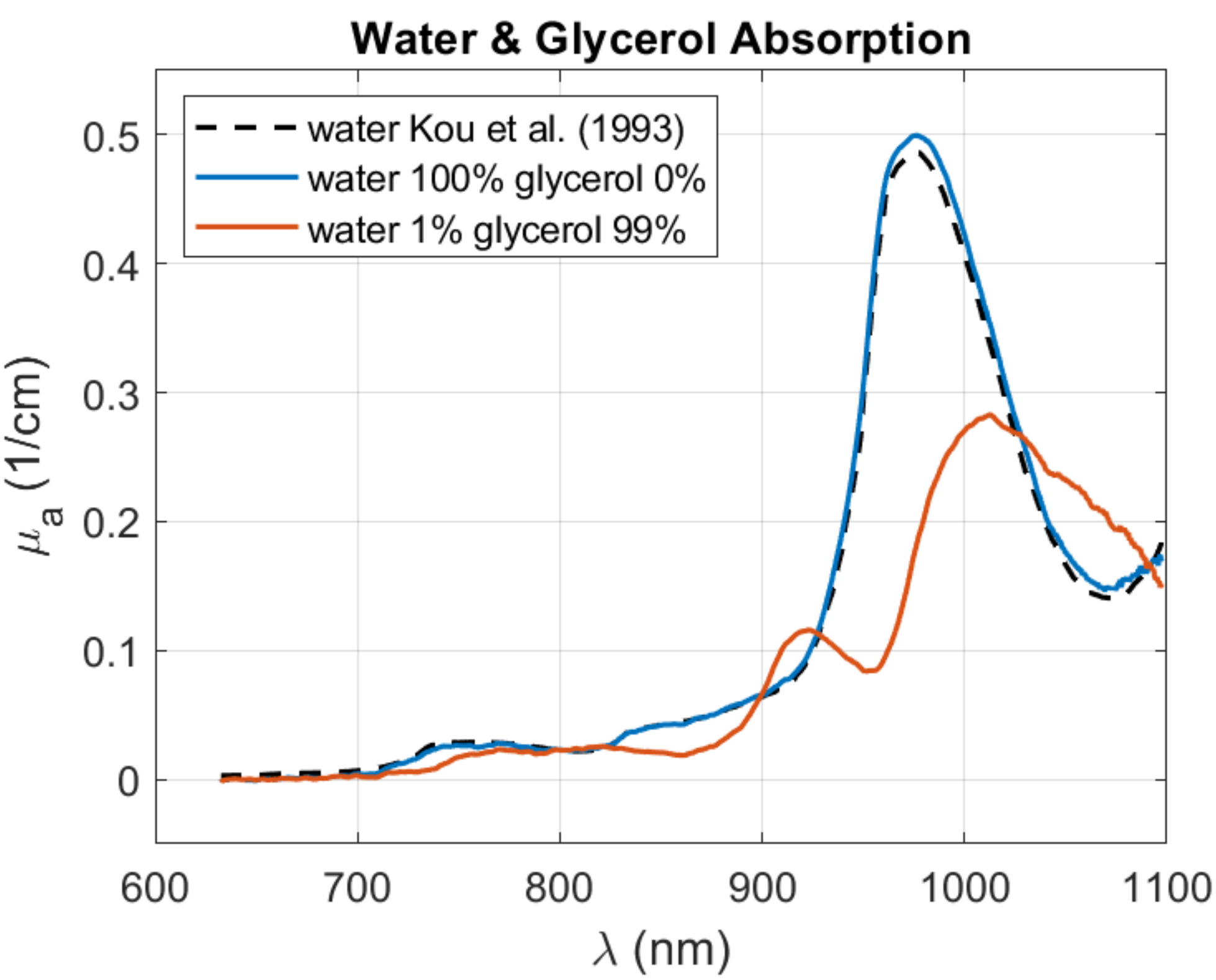

Fig. S5. Near-infrared absorption coefficient spectra over 640–1100 nm. The dashed black trace shows the literature water absorption spectrum from Kou et al. (1993), while the solid blue and orange traces show measured absorption spectra of water and commercially supplied glycerol (99%; PTI Process Chemicals, United States), respectively.

S9 Observation of scattering drift during phantom deoxygenation

As mentioned in Sec. 3.3, during the deoxygenation phase of the blood-yeast phantom measurement, there were changes to the measured scattering values as assessed by FD-NIRS. These changes can be seen to be progressive as the hemoglobin saturation transitions from 100% to 0% (Fig. S6(a)), which results in a change in the overall shape of the reduced scattering spectrum. Fitting the spectrally resolved reduced scattering coefficients at each time point throughout the deoxygenation phase reveals a progressive decrease in the scattering amplitude (*a*) and a decrease in magnitude of the scattering power (*b*) (Fig. S6(b)).

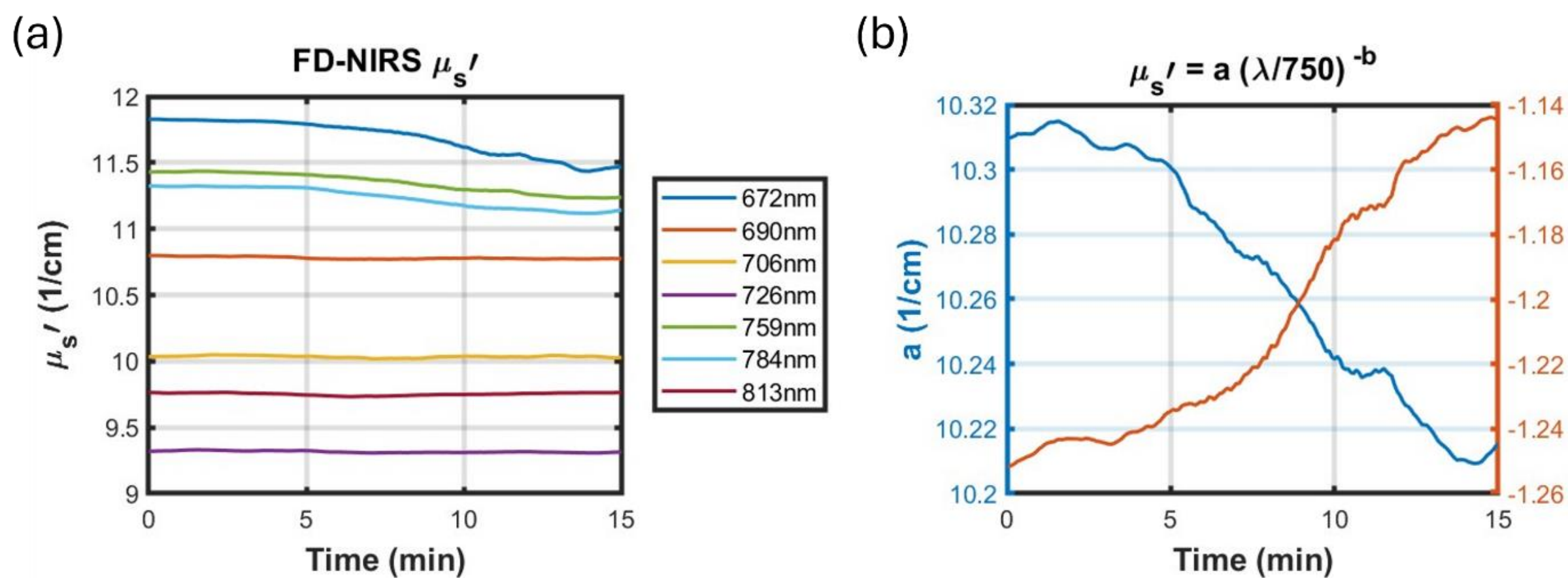

Fig. S6 Changes in reduced scattering coefficients during deoxygenation in the yeast-blood phantom measured with MetaOx, the FD-NIRS device: (a) reduced scattering coefficients at each wavelength; (b) resulting a and b parameters of power-law fitting, using λ_0 = 750 nm.

S10 Correlations between tissue chromophore concentration changes during breathing tasks

To investigate the possible source of the fluctuations in the water signal during the breathing tasks, in Fig. S7, we compare the correlation coefficients of the changes in the other chromophore signal traces (ΔHbO, ΔHbR, ΔHbT) to the signal trace of the water signal (ΔH_2O). As discussed in Sec. 3.5, the changes in the water signal were most correlated with changes in oxygenated hemoglobin

and total hemoglobin. We hypothesize these results indicate the sensitivity of the changes in the water signal to changes in blood volume, which we anticipate given the arterial vasodilation and vasoconstriction induced by the breathing tasks.

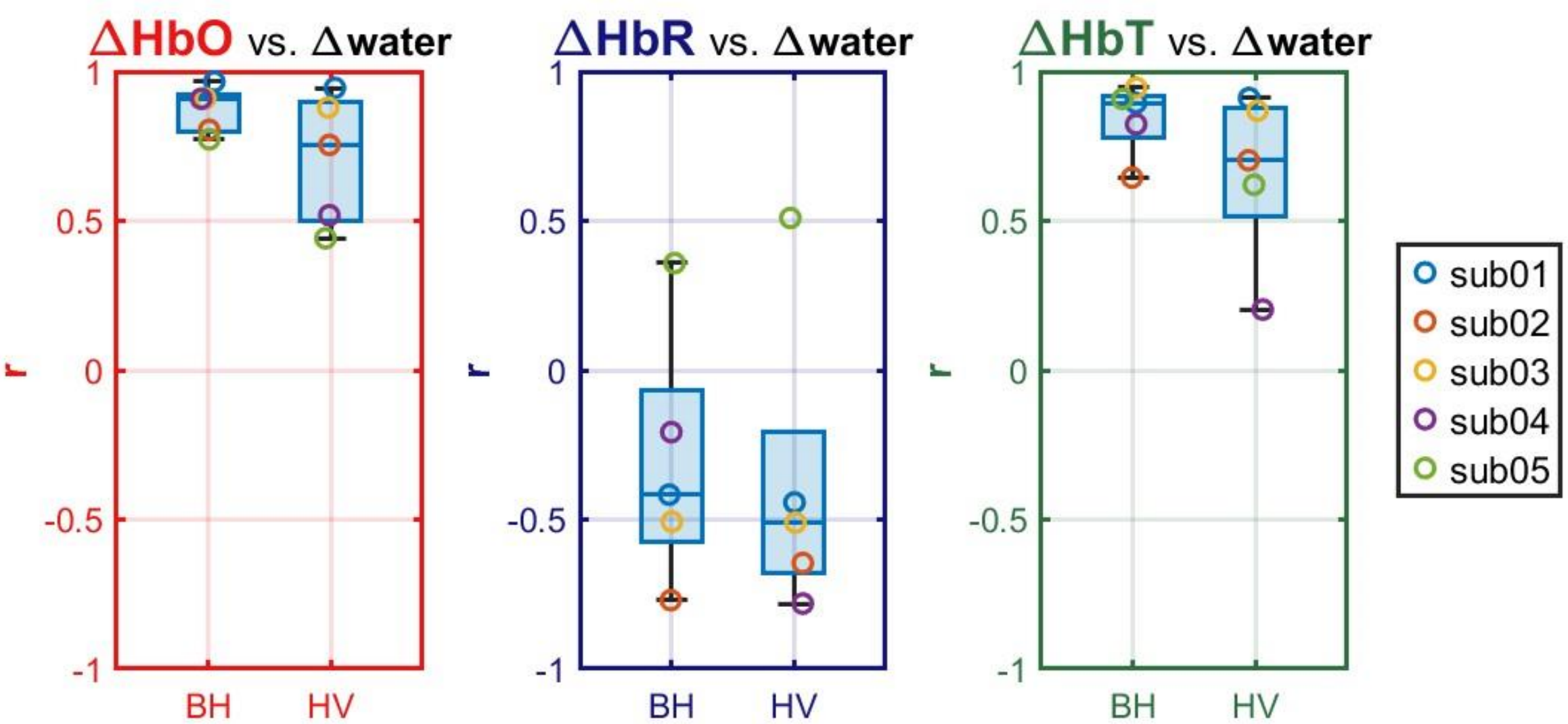

Fig. S7 Pearson correlation coefficients (r) between changes in water fraction (ΔH_2O) and changes in oxyhemoglobin (ΔHbO), deoxyhemoglobin (ΔHbR), and total hemoglobin (ΔHbT), shown separately for breath-holding (BH) and hyperventilation (HV). Box plots summarize the distributions across participants, and identically colored markers denote the same participant across maneuvers.

References

1. N. Otic et al., "MW-FlexNIRS: wearable, low-cost, LED-based, multi-wavelength NIRS oximeter for cytochrome c oxidase recovery in neonates," Biomed. Opt. Express **17**(4), 1649–1672, Optica Publishing Group (2026) [doi:10.1364/BOE.590418].
2. F. Scholkmann et al., "A review on continuous wave functional near-infrared spectroscopy and imaging instrumentation and methodology," Neuroimage **85**, 6–27, Elsevier (2014) [doi:10.1016/j.neuroimage.2013.05.004].
3. H. Liu et al., "Simplified approach to characterize optical properties and blood oxygenation

in tissue using continuous near-infrared light," in Optical Tomography, Photon Migration, and Spectroscopy of Tissue and Model Media: Theory, Human Studies, and Instrumentation **2389**, pp. 496–502, SPIE (1995) [doi:10.1117/12.209998].

4. S. Fantini et al., "Non-invasive optical monitoring of the newborn piglet brain using continuous-wave and frequency-domain spectroscopy," Phys. Med. Biol. **44**(6), 1543–1563 (1999) [doi:10.1088/0031-9155/44/6/308].

5. T. J. Farrell, M. S. Patterson, and B. Wilson, "A diffusion theory model of spatially resolved, steady-state diffuse reflectance for the noninvasive determination of tissue optical properties in vivo," Med. Phys. **19**(4), 879–888, Wiley Online Library (1992) [doi:10.1118/1.596777].

6. S. L. Jacques, "Optical properties of biological tissues: a review," Phys. Med. Biol. **58**(11), R37–R61 (2013) [doi:10.1088/0031-9155/58/11/R37].

7. ASTM International, "ASTM C1055-20, Standard Guide for Heated System Surface Conditions that Produce Contact Burn Injuries," ASTM International, West Conshohocken, PA (2020) [doi:10.1520/C1055-20].

8. L. Kou, D. Labrie, and P. Chylek, "Refractive indices of water and ice in the 0.65- to 2.5-μm spectral range," Appl. Opt. **32**(19), 3531–3540, Optical Society of America (1993) [doi:10.1364/AO.32.003531].
